# A reversal of Glagov's hypothesis; a preliminary demonstration by cardiac magnetic resonance

**DOI:** 10.1186/1532-429X-13-S1-P178

**Published:** 2011-02-02

**Authors:** Sobhan Kodali, Mark Doyle, Saundra B Grant, David R Neff, Ronald B Williams, June A Yamrozik, Geetha Rayarao, George Angheloiu, Vikas K Rathi, Robert WW Biederman

**Affiliations:** 1 Allegheny General Hospital, Pittsburgh, PA, USA; 2 Bon Secours Heart and Vascular Institute, Richmond, VA, USA

## Introduction

According to Glagov's hypothesis, the early stages of atherogenesis (stenosis <40%) are associated with positive remodeling of the outer arterial wall resulting in paradoxical preservation of the luminal area, and as stenosis exceeds 40%, further plaque accumulation results in a decrease in the area of the lumen. This study examines the effects of statins on human carotid atherosclerotic plaque to investigate if a reversal of Glagov's hypothesis occurs with statin therapy.

## Purpose

We proposed that Glagov's hypothesis will work in reverse upon institution of statin therapy as observed with high-resolution Cardiac Magnetic Resonance (CMR).

## Methods

Via CMR (1.5T GE, WI), 35 carotid arteries of 18 asymptomatic, ‘statin naïve’ patients with maximum carotid stenosis >50%, were imaged at baseline and following 12 months of randomized statin therapy (simvastatin 40mg or Vytorin (Ezetimibe 10mg/ simvastatin 40mg)). The effects of statins on these lesions were evaluated as changes in carotid outer wall area (OWA), lumen area (LA), vessel wall area (VWA), lipid area (LpA) and lipid percentage (Lp%). The percentage of stenosis of each slice was determined in reference to a normal or near normal slice in the corresponding carotid artery. Plaque morphology was determined by T1 and T2/PD. Mean resolution was 1x1x2mm.

## Results

706-2mm slices were available: 265 slices with < 40% and 441 slices with > 40% stenosis prior to and after 12 months of statin therapy. Slices with < 40% showed a significant decrease in OWA (118.8 versus 107.21 mm^2^; P< .001), VWA (82.9 versus 76.7 mm^2^; P=0.004), LpA (25.7 versus 23.7 mm^2^; P=0.04) and LA (35.8 versus 30.8 mm^2^; P<0.001). Lp% remained unchanged (30% versus 29.6%). Among slices with >40% stenosis, there was no significant change in any of the parameters studied. There was also no statistically significant change in any of the parameters when all contiguous slices of all carotid plaques were analyzed in a 3D fashion.

## Conclusions

Changes in carotid plaque after lipid lowering therapy, as measured by high-resolution CMR depict a reversal of Glagov's hypothesis *only* in areas of <40% stenosis; i.e, a decrease in the area of the outer wall, vessel wall and lipid portion with a concomitant but unexpected decrease in luminal area.

